# Cost-Effectively 3D-Printed Rigid and Versatile Interpenetrating Polymer Networks

**DOI:** 10.3390/ma14164544

**Published:** 2021-08-12

**Authors:** Osman Konuray, Arnau Sola, Jordi Bonada, Agnieszka Tercjak, Albert Fabregat-Sanjuan, Xavier Fernández-Francos, Xavier Ramis

**Affiliations:** 1 Thermodynamics Laboratory, ETSEIB, Universitat Politècnica de Catalunya, Avda. Diagonal 647, 08028 Barcelona, Spain; arnisola@gmail.com (A.S.); xavier.fernandez@upc.edu (X.F.-F.); xavier.ramis@upc.edu (X.R.); 2 Department of Strength of Materials and Structural Engineering, ETSEIB, Universitat Politècnica de Catalunya, Avda. Diagonal 647, 08028 Barcelona, Spain; jordi.bonada@upc.edu; 3 Group ‘Materials + Technologies’, Department of Chemical Engineering and Environment, Universidad País Vasco/Euskal Herriko Unibertsitatea, Pza. Europa 1, 20018 Donostia-San Sebastián, Spain; agnieszka.tercjaks@ehu.eus; 4 Department of Mechanical Engineering, Universitat Rovira i Virgili, Av. Països Catalans 26, 43007 Tarragona, Spain; a.fabregat@urv.cat

**Keywords:** interpenetrating polymer network, dual-curing, digital light processing

## Abstract

Versatile acrylate–epoxy hybrid formulations are becoming widespread in photo/thermal dual-processing scenarios, especially in 3D printing applications. Usually, parts are printed in a stereolithography or digital light processing (DLP) 3D printer, after which a thermal treatment would bestow the final material with superior mechanical properties. We report the successful formulation of such a hybrid system, consisting of a commercial 3D printing acrylate resin modified by an epoxy–anhydride mixture. In the final polymeric network, we observed segregation of an epoxy-rich phase as nano-domains, similar to what was observed in a previous work. However, in the current work, we show the effectiveness of a coupling agent added to the formulation to mitigate this segregation for when such phase separation is undesired. The hybrid materials showed significant improvement of Young’s modulus over the neat acrylate. Once the flexible, partially-cured material was printed with a minimal number of layers, it could be molded into a complex form and thermally cured. Temporary shapes were readily programmable on this final material, with easy shape recovery under mild temperatures. Inspired by repairable 3D printed materials described recently, we manufactured a large object by printing its two halves, and then joined them covalently at the thermal cure stage with an apparently seamless union.

## 1. Introduction

The imminent advent of the fourth industrial revolution has put environmentally-friendly, low volume, mass-customization manufacturing processes on a pedestal, as the success of the revolution depends on sustainable and quickly adaptable techniques. In this sense, additive manufacturing (or more popularly, 3D printing) is garnering increased attention from various industry fields, as it facilitates more flexible product design processes in comparison to conventional manufacturing methods [1,2]. Different 3D printing techniques are being adopted at a very fast pace by industries such as biomaterials [3], construction [4], and sportswear [2], to name just a few. As the general industry becomes less and less mass production oriented, custom manufacturing methods that provide speed and flexibility are increasingly favored. More and more research is being published on 3D printing materials and methods, intended for industrial use, evidencing their undeniable potential in replacing conventional production practices. Among the various methods, photocure-based methods such as stereolithography and digital light processing (DLP) stand out with fast production rates, simpler workflow and easy-to-use equipment. Nevertheless, there are many obstacles to overcome, especially at the material level, before we can witness a complete takeover by these photocure systems. Whereas purely acrylate based photopolymerization processes suffer from oxygen inhibition—except when such inhibition is essential in certain configurations such as continuous liquid interface production (CLIP)—purely epoxide systems, based on cationic photopolymerization, suffer from low curing rates and dark curing [5,6,7]. The latter problem oftentimes leads to loss of resolution in printed materials. Hybrid (or dual-cure) formulations combine the advantages of both free radical and cationic (or base/nucleophile initiated) polymerizations, while alleviating their common problems, such as dark-curing, which can actually prove beneficial in achieving higher conversions after manufacture. Numerous hybrid systems have been described in recent literature. In all these works, researchers employed dual-curing approaches wherein the desirable kinetics of free radical acrylate photopolymerization is coupled with the high rigidity of epoxy-based polymeric networks. Whereas in some works the free radical acrylate photopolymerization took place concurrently with cationic epoxy curing [8,9,10,11,12,13], in others, a sequential dual-process was used in which the epoxy was cured in a thermal step, subsequent to acrylate photopolymerization [14,15,16,17]. Very recently, our group has published a comprehensive review on the recent state-of-art of dual-processing of 3D printing materials [18].

In this work, a sequentially dual-cured hybrid acrylate–epoxy system is presented. We use a commercial photoresin formulation named Spot-E, which contains urethane acrylate oligomers and aliphatic acrylates, and is intended for applications requiring certain elasticity in printed parts (hence the extension “E”). We modify this resin with an epoxy–anhydride compound cured by a nucleophilic initiator. Given that the remaining acrylates are difficult to cure merely by UV post curing, especially if the part is thick and/or contains fillers [19], the formulation also contains a thermally activated radical generator, which ensures all unreacted acrylates remaining from the printing stage are fully cured concurrently with epoxy–anhydride copolymerization. The relative contribution of the epoxy–anhydride network and the thermal radical initiator to the ultimate mechanical performance will be shown. As will also be shown, by varying the relative amounts of acrylate and epoxy in the formulation, a selection of materials with a wide range of thermomechanical properties could be obtained. Once the epoxy–anhydride copolymerization takes place, in certain formulations, the formed network formed a separated phase, evidenced by dynamic mechanical analysis (DMA) and atomic force microscopy (AFM). Although such separation was observed previously in similar systems [17,20], its effective mitigation was not attempted. In this work, by employing a coupling agent bearing carboxyl and acrylate groups, the separation could be suppressed. Thanks to the thermal radical initiator, our hybrid system can be cured in a purely thermal process. In this case, a careful selection of curing temperatures would be necessary in order to ensure control of the curing sequence and its kinetics.

In contrast to our previous work, the use of Spot-E resin A yields rubbery parts that are flexible and thus shape-conformable after 3D printing at ambient temperature [17,20]. This flexibility can be exploited from an economical point of view, by printing simple 2D geometries in significantly fewer layers, and thus in a shorter time. These printed intermediate materials can later be formed in complex 3D shapes and thermally cured to fix the shape. Thanks to their intrinsic shape memory capability, these complex shapes can be temporarily programmed into any arbitrary form in a reversible fashion, as per transport and processing needs, through appropriate heating–cooling cycles. Prospectively, the dual-cure capability can be exploited to covalently bind printed parts to produce objects larger than the printing platform. After printing the parts, they can be joined and thermally treated to form covalent bonds at the interface. Based on this idea and inspired by recently described repairable 3D printed materials, in this work we also demonstrate the manufacture of a large object by parts. Whereas in some of these recent publications the repair is achieved through bond exchange reactions [21,22,23], in others it is achieved through new bond formation [24], similar to what we demonstrate.

## 2. Materials and Methods

The commercial photoresin Spot-E (Spot-A Materials), which will be denoted simply as E hereafter, is a mixture of urethane–acrylate oligomers and other aliphatic acrylate monomers (the exact composition is proprietary information), with an average molecular weight of M = 627 g/mol and functionality f = 1.32. The preparation contains photoinitiators and photoinhibitors at adequate amounts to balance the curing rate and printing resolution, and the photocure can be initiated at UV or visible wavelengths.

The epoxy component is a diglycidyl ether of Bisphenol-A (tradename EBL70, Po. int. Er. S.r.l., Asti, Italy), coded as DG, with an equivalent weight of 184.5 g per epoxy group. The anhydride curing agent is hexahydro-4-methylphtalic anhydride (Sigma-Aldrich, Madrid, Spain), coded as MA, with an equivalent weight of 168.19 g per anhydride group. The epoxy-anhydride mixture is denoted simply as DGMA. A nucleophilic initiator, 4-(dimethylamino)pyridine (DMAP) (Sigma-Aldrich, Madrid, Spain) is used to catalyze the epoxy–anhydride copolymerization. The thermally initiated radical generator is a 60% solution of d’1,1-di(tert-amil peroxy)-cyclohexane, with tradename LUPEROX 531M60 (abbreviated as LUP), and was provided by ARKEMA. At the thermal curing stage, this initiator ensures complete conversion of all unreacted acrylates remaining from the DLP-3D printing stage. For a more detailed discussion of its function, the reader is directed to our earlier work [17,20]. Although present also in the FTIR samples, the initiator is essentially redundant, since the UV irradiation conditions in the FTIR spectrometer ensure full conversion of acrylates at ambient temperature, as will be shown later. The coupling agent (denoted as CA) is 2-carboxyethyl acrylate oligomer with a molecular weight of 170 g/mol acid or acrylate (Sigma-Aldrich, Madrid, Spain). The epoxy resin DG was dried in a vacuum oven at 80 °C for 2 h prior to use. The other reagents were used as received.

The dual formulations were prepared in the following manner: The acrylate and epoxy components were prepared separately and then mixed in predetermined proportions. For the acrylate part, LUP was taken to a glass vial wrapped in aluminum foil or to an opaque container and Spot-E was added on top. The weight ratio was LUP/E = 0.25/99.75. In a separate glass vial, DG and MA were mixed stoichiometrically: 1 epoxy equivalent of DG (184.5 g/mol) per anhydride group (168.2 g/mol). DMAP (shortened as P in coding certain formulations) was added so that the weight ratio DMAP/DGMA was 0.025/99.975. The coupling agent CA was incorporated in combination with extra DG in a stoichiometric proportion (hereafter coded as DGCA): 1 epoxy equivalent of DG (184.5 g/mol) per acid group (170 g/mol).

The dual formulations were denoted as ExDGMAyDGCAz, where x and y are weight percentages of E (including LUP), DGMA (including DMAP), and DGCA, respectively. The composition of the different formulations in E, CA, DG, and MA (wt.%) is indicated in Table 1.

A Mettler DSC3+ refrigerant cooled thermal analyzer (Mettler Toledo) was used for thermal *T_g_* and residual polymerization heat measurements. A Brucker Vertex 70 (Bruker Optics Inc., Billerica, MA, USA) equipped with an attenuated total reflection (ATR) accessory (GoldenGate™) (Specac Ltd., Orpington, UK) and temperature control unit was used to monitor reactive group conversions. For the photocuring stage, the absorption band at 1730 cm^−1^ corresponding to C=O carbonyl stretching of the ester groups was used as reference. For the epoxy–anhydride curing, it was the band corresponding to C–H stretching at 2965–2850 cm^−1^. The epoxy band at 894 cm^−1^ was analyzed only qualitatively, since this is a convoluted band. Out of the three acrylate bands (i.e., 810, 1400, or 1636–1620 cm^−1^) available for quantitative analysis, the band at 1400 cm^−1^ was used. Although not shown, the other two bands gave practically identical results. In both DSC and FTIR instruments, a Hamamatsu Lightningcure LC5 (Hg–Xe lamp, irradiation intensity < 2 mW·cm^–2^) was used for the photocuring stage. We analyze the obtained data by DSC and FTIR in the same way, as explained in our previous works [20,25].

Dynamic mechanical analysis (DMA) was used to obtain storage modulus and tan delta curves of intermediate and final materials. For viscoelastic materials under a given stress or strain load, storage (*E’*) and loss moduli (*E”*) represent the stored energy (elastic part) and the energy dissipated as heat (viscous part), respectively. tan delta is simply *E”/E’*. DMA was performed with a TA Instruments DMA Q800 device (TA Instruments, New Castle, DE, USA) using single cantilever clamps at a frequency of 1 Hz and 0.05% strain, in a sufficiently wide temperature range to fully observe network relaxation. The heating rate in all DMA scans was 3 °C/min.

Atomic force microscopy (AFM) was used to study nano-scale morphology of the final materials. The equipment used was a scanning probe microscope (SPM) (NanoScope IIIa Multimode from Digital Instruments, Veeco Instruments Inc., Plainview, NY, USA) in tapping mode (TM-AFM). One beam cantilever (125 mm) with a silicon probe (curvature nominal radius of 5–10 nm) was used. Samples were cut using an ultramicrotome Leica Ultracut R with a diamond blade.

Compression tests were performed using an Instron 3366 Universal testing Machine (Instron, Barcelona, Spain), according to the ASTM D695-15 standard. The nominal dimensions of the test specimen were 12.7 × 12.7 × 50.8 mm^3^. The test was performed at a constant displacement rate of 2.6 mm/min until specimen failure or equipment limit (10 kN).

Tensile tests were performed using the same Instron 3366 universal testing machine. Dog-bone shaped specimens were analyzed with dimensions according to the ASTM D638-14 standard; type IV specimens were tested that had an overall length and overall width of 115 and 19 mm, respectively. The test was performed at a constant displacement rate of 2 mm/min until specimen failure. The displacement rate was adjusted to obtain the same strain rate as compression tests in order to be able to compare moduli results with those obtained by compression tests.

The samples for DMA and AFM analyses were prepared by UV flood curing in a Vilber Lourmat UV oven (4 mW/cm^2^, 365 nm wavelength) followed by thermal postcuring 3 h at 150 °C in a Memmert convection oven. The liquid formulations were poured into rectangular prismatic molds of 20 × 10 × 1 mm^3^ made with Teflon and covered with glass slides to enable penetration of UV light. The samples were UV-cured by alternative short exposures on both sides of the sample, with a total dose of 2 J/cm^2^. The UV-cured samples were demolded prior to thermal curing.

An Asiga MAX UV desktop 3D printer was used to print the different samples for mechanical analysis and assembly/shape memory demonstration tests. Each sample was designed using FreeCAD software and exported in STL file format, and the file was subsequently uploaded to the printer. Average wavelength of irradiation was 385 nm, with an irradiation intensity of 6 mW/cm^2^. Layer thickness was fixed at 100 um. Each layer received an irradiation of 17 mJ/cm^2^, which was an optimal dose as per calibration results. The printed samples were thermally postcured at 150 °C for 3 h.

## 3. Results and Discussion

### 3.1. Curing Kinetics

**Scheme 1:**
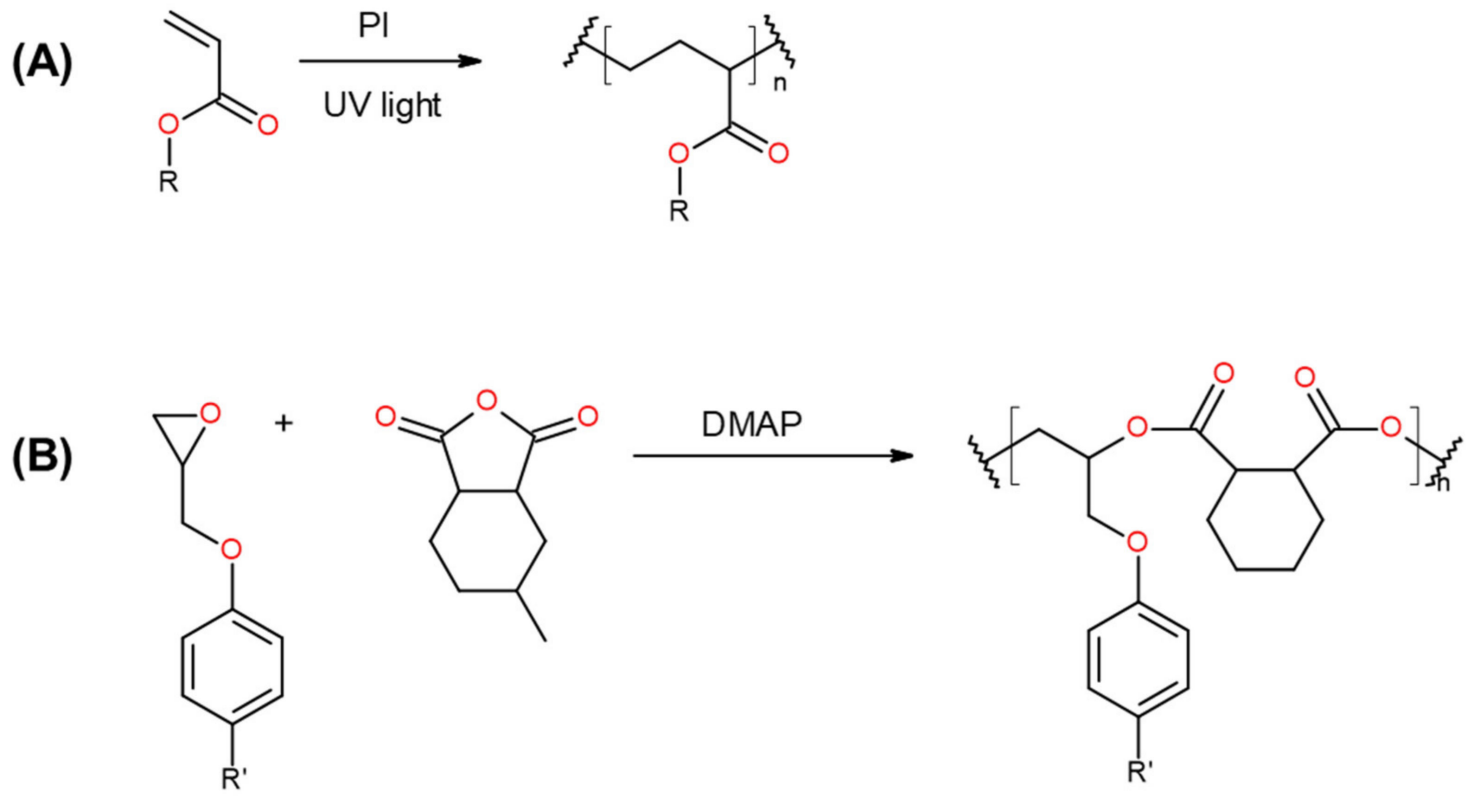
(**A**) Polyacrylate network formed by photocuring using a photoinitiator and (**B**) copolyester network formed by the epoxy–anhydride copolymerization thermally activated using DMAP as initiator.

As explained previously, the first curing stage is free-radical acrylate photopolymerization at ambient temperature and the second curing stage is nucleophilically initiated epoxy–anhydride copolymerization carried out at elevated temperature. The overall reaction process is similar to that reported in our previous works [17,26]. A polyacrylate network is formed by radical acrylate homopolymerization initiated by activation of a photoinitiator upon exposure to UV light, and a copolyester network is formed by epoxy–anhydride copolymerization by initiation with a nucleophilic tertiary amine such as DMAP, as seen in Scheme 1. The initiation by DMAP follows the mechanism explained elsewhere [27].

**Scheme 2:**
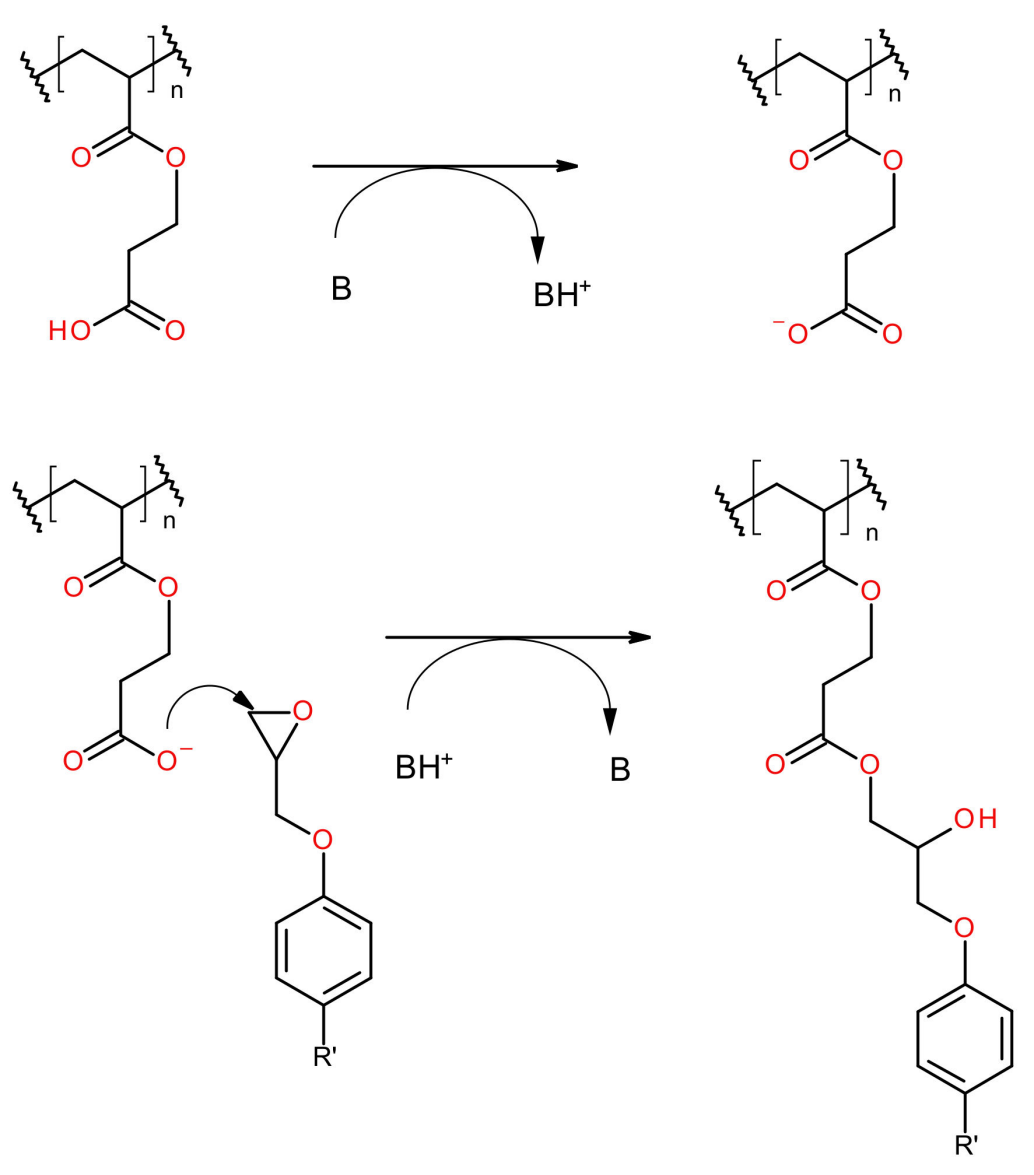
The coupling of the poly(acrylate) and the poly(epoxide) networks through a base/nucleophile-initiated mechanism. Any basic species present in the reaction medium can effectively mediate the deprotonation. The CA is shown here as pre-incorporated into the poly(acrylate) network. B: Base.

In the present work we employed a coupling agent to covalently bind the two polymeric networks. This acrylate-functional coupling agent bearing an acid group is first incorporated into the polyacrylate network structure (Scheme 1A). The existence of the pendant acid group enables the formation of a covalent bond between the acrylate and epoxy networks, with the underlying mechanism depicted in Scheme 2. A base such as DMAP or other species such as carboxylate anions present in the epoxy–anhydride copolymerization [28,29] can deprotonate the CA, forming a new carboxylate anion. This anion, in turn, can attack an epoxy group, as illustrated in Scheme 2. The presence of this coupling agent and its participation in the second curing stage will therefore have a significant influence in the final morphology and properties of the cured networks, as will be shown later.

Keeping the overall reaction scheme in mind, in this section we only provide a concise description of the dual-curing kinetics of the CA-free E40DGMA60 that was obtained using DSC and FTIR. Among the different dual formulations tested, this particular one achieves the best balance between ductility after 3D printing and rigidity after thermal treatment.

**Figure 1:**
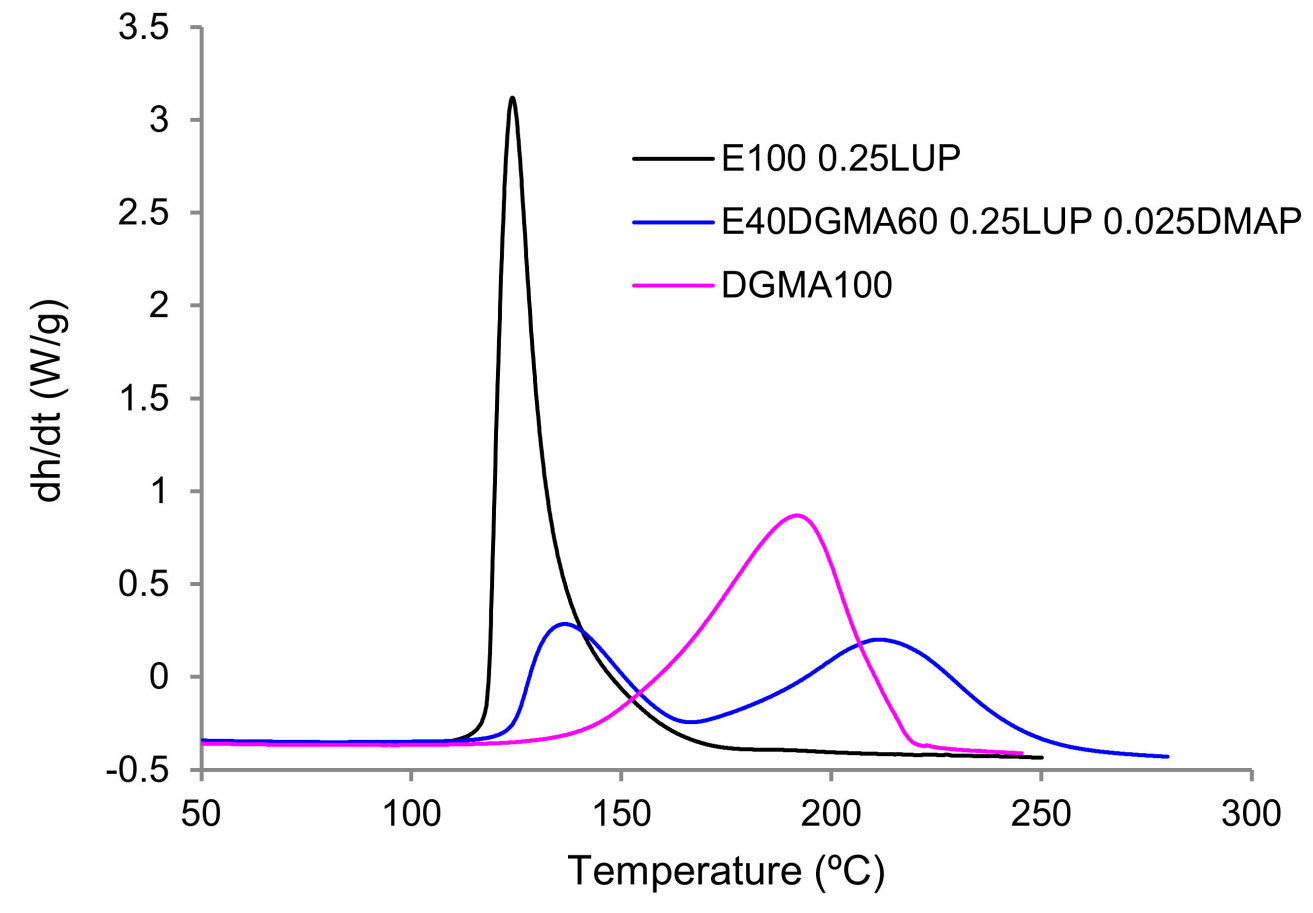
DSC heat flow curing curve of the dual formulation E40DGMA60 juxtaposed with each of its curing stages carried out separately.

Figure 1 shows the DSC heat flow curves of three samples: the neat acrylate resin with LUP, the dual formulation E40DGMA60 with both LUP and DMAP, and the pure epoxy–anhydride (DGMA100) with DMAP. A purely thermal curing scenario is tested here in order to illustrate the sequential dual-curing character of the formulation. As can be seen, a well-defined curing sequence is obtained, the acrylate homopolymerization taking place firstly, followed by the epoxy–anhydride copolymerization. It is observed, though, that both curing stages of E40DGMA60 start later, in comparison to the neat components. Whereas the slowing of stage 1 is due to the dilution by the epoxy–anhydride component, the slowing of stage 2 is due to mobility restrictions posed by the poly(acrylate) network formed. One can argue that DMAP might have favored the redox composition of LUP, which would have accelerated the radical formation as stated in a previous work [26]. However, in the present work a glycidyl epoxy resin was used instead of a cycloaliphatic one. As a consequence, the effect of DMAP on the redox activation of LUP might be less relevant due to the formation of a different species by interaction between DMAP and the epoxy group [27]. This analysis also evidences that the epoxy–anhydride reaction only takes place at elevated temperatures and therefore it is possible to establish a safe dual-curing process which we can use to our advantage in 3D printing. The dual process consists of (1) a room-temperature photocuring stage in the 3D-printer consisting of acrylate homopolymerization followed by (2) a thermally activated epoxy–anhydride copolymerization at an elevated temperature in a conventional oven. In addition, these systems can also be processed in a sequential way using only thermally controlled activation of the different reactions if desired. A more detailed kinetic analysis of this dual-curing system has recently been presented by our research group [30].

**Figure 2:**
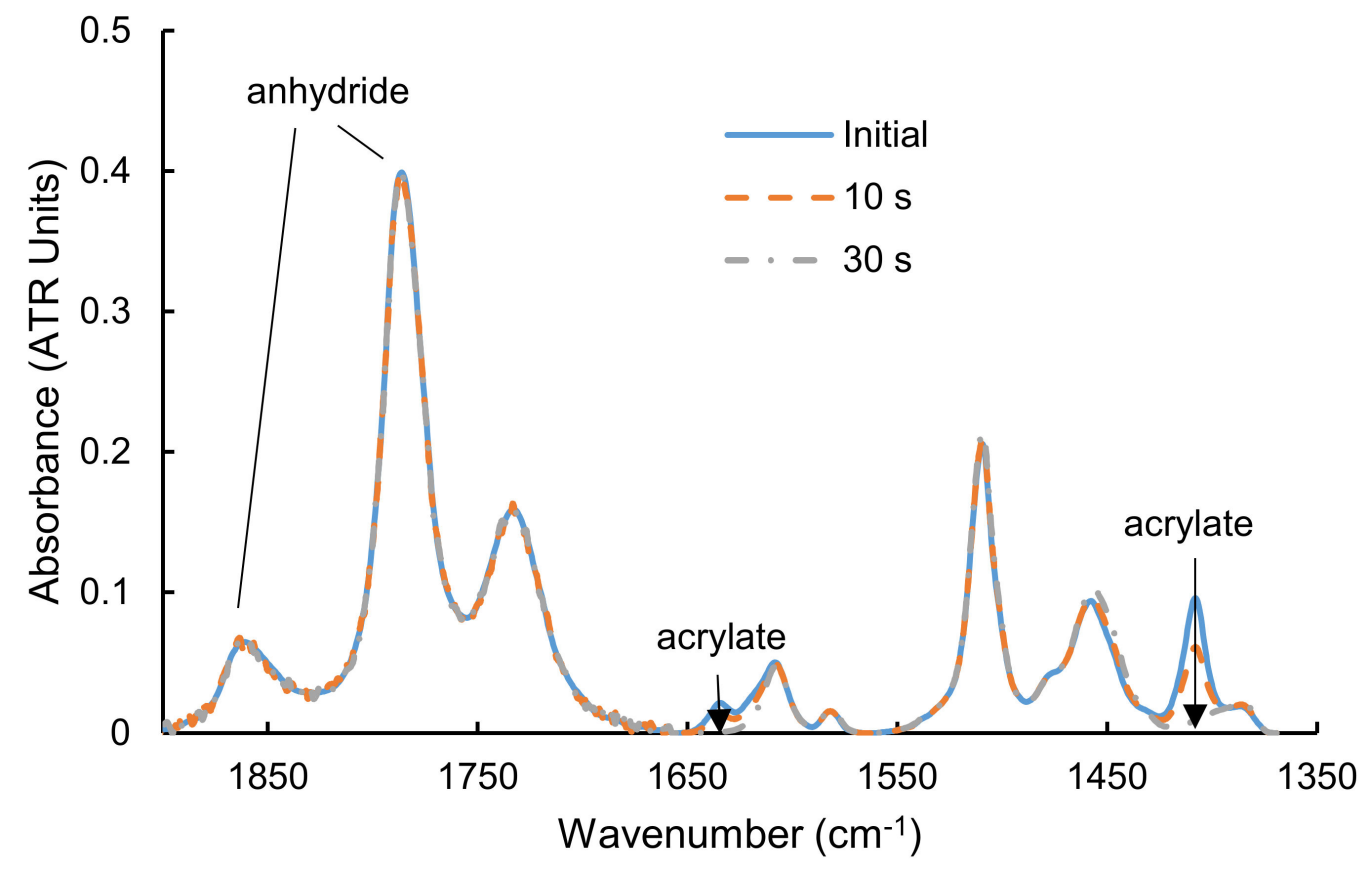
Acrylate absorption bands disappear in 30 s of photocure of E40DGMA60. Anhydride bands remain unchanged, confirming that no epoxy–anhydride reaction takes place.

**Figure 3:**
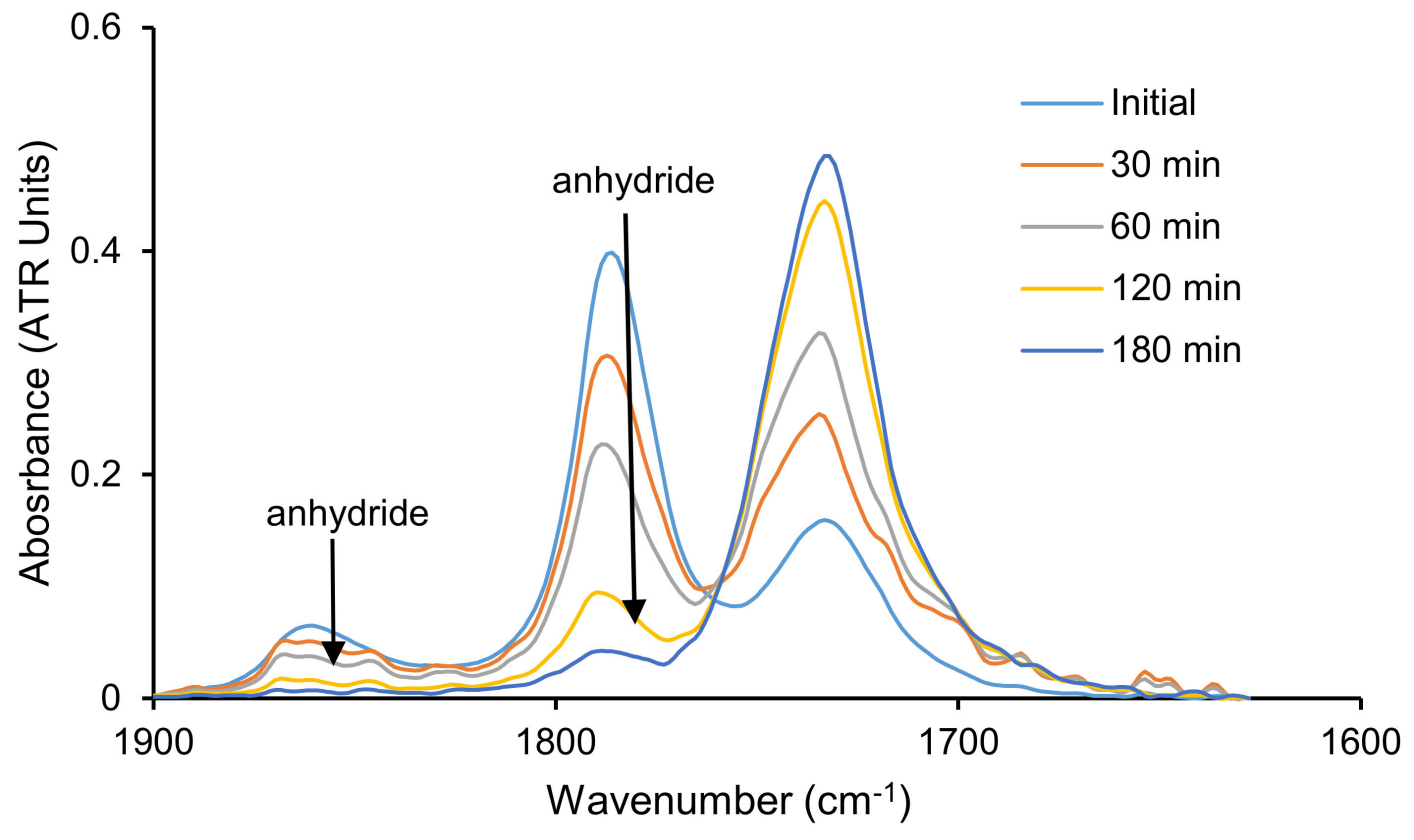
Anhydride absorption bands disappear after 3 h of thermal treatment at 150 °C. The formulation is E40DGMA60.

In Figure 2 and Figure 3 we show FTIR spectra during stage 1 (photo) curing, and during stage 2 (thermal) curing, respectively. Stage 2 temperature was chosen as 150 °C based on DSC results discussed previously. As can be seen, even at such low light intensities (<2 mW cm^−2^), the acrylate groups are virtually depleted in 30 s. As anhydride bands remain unchanged, it is confirmed that no epoxy–anhydride reaction takes place. Once the temperature is raised to 150 °C, the epoxy–anhydride reaction commences with complete disappearance of anhydride bands after 3 h (Figure 3). This result establishes the stage 2 curing parameters for the remainder of the work.

With regard to storage stability of the liquid preparations, it was confirmed that *T_g_* and residual heats were unchanged after the first week of storage at 25 °C, with only a slight increase in viscosity after the first month, which still did not impede their 3D printability. These results are in sync with our earlier report on a similar dual system [17].

### 3.2. Material Properties

#### 3.2.1. Thermal and Viscoelastic Properties

As reported in previous works [20], the intermediate materials, obtained after the first photocuring or 3D-printing stage, are gelled due to the formation of the polyacrylate network. The intermediate materials were soft and ductile due to the inherent flexibility of the Spot-E polyacrylate network and the plasticization by the liquid epoxy–anhydride mixture. After the second curing stage, a significant increase in rigidity was observed due to the formation of the epoxy–anhydride network structure, which was not unexpected due to the high *T_g_* and rigidity of the DGMA network [31]. Both the intermediate and final materials appeared to be macroscopically uniform.

**Figure 4:**
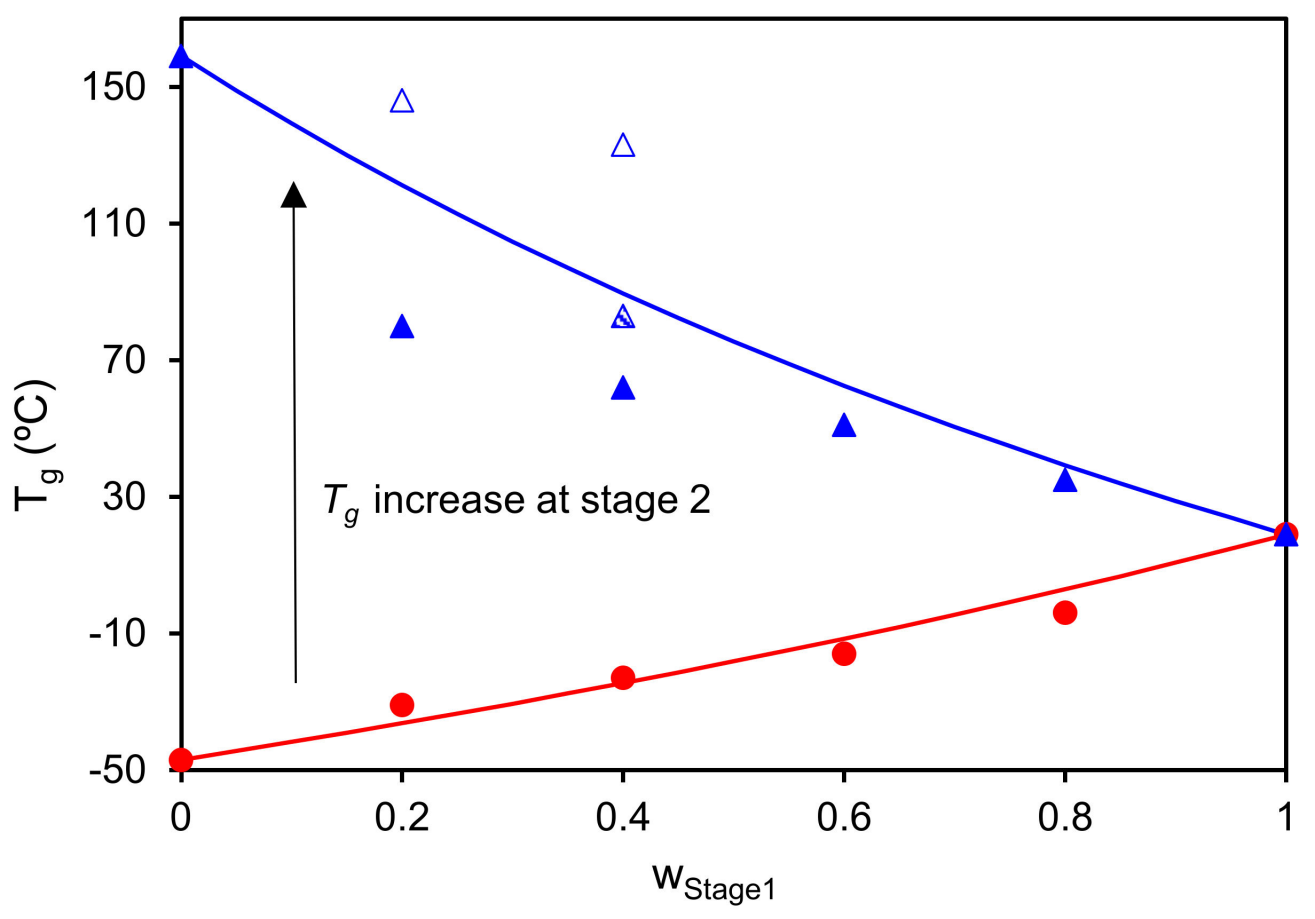
Intermediate (circles) and final (triangles) *T_g_* determined by DSC (or taken as loss moduli peaks for E20DGMA80 and E40DGMA60) as functions of acrylate component weight fraction. Lines represent theoretical *T_g_* calculated by the Fox equation. The singular *T_g_* of the CA containing E32.5DGMA52.5DGCA15 formulation is shown as a shaded triangle.

In Figure 4 we provide a range of DSC *T_g_* values of our materials at the intermediate (circles) and final stage (triangles), as a function of acrylate component weight percentage. Also shown in the figure are the theoretical *T_g_* values (lines) calculated by the well-known Fox expression given in Equation (1) [32].

(1) $$\frac{1}{T_{g}}=\frac{w_{1}}{T_{g,1}}+\frac{\left(1-w_{1}\right)}{T_{g,2}}$$

As can be seen, experimental *T_g_* fit well with the predicted values at the intermediate stage, due to the good compatibility between the acrylate network and the unreacted epoxy and anhydride monomers. At the final stage, however, especially in epoxy-rich formulations, experimental results deviate from predictions. The final *T_g_* values were impossible to quantify with DSC, as the heat capacity steps were barely perceptible. Nevertheless, our DSC analysis suggested secondary relaxations, which were corroborated by DMA analysis. Since DMA loss moduli peaks can be taken as fair estimates of DSC *T_g_*, we plotted these as experimental *T_g_* on the figure. The appearance of two relaxation peaks in the tan δ and loss moduli traces suggests phase separation.

**Figure 5:**
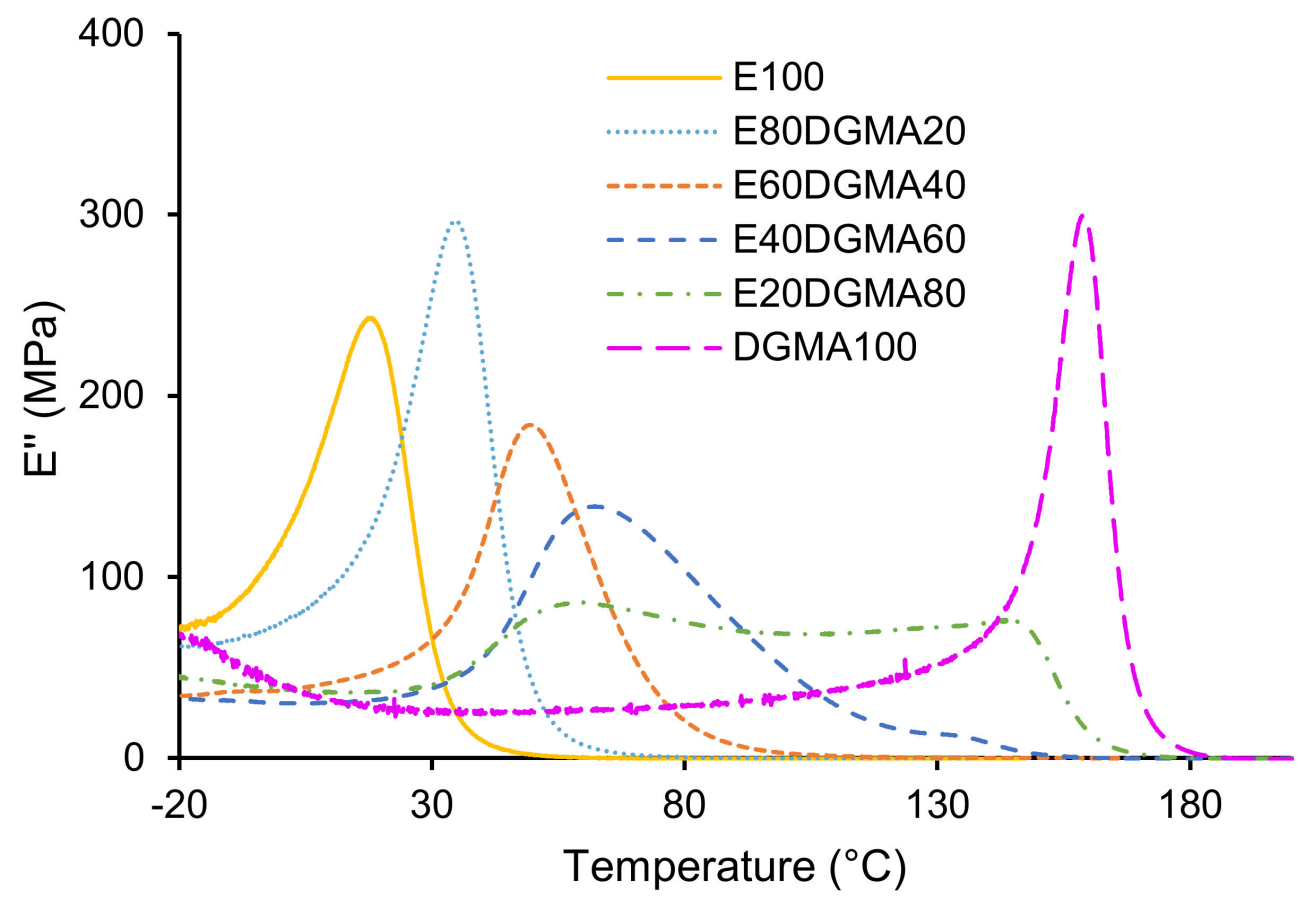
Loss modulus ($E^{\prime \prime }$) curves of all ExDGMAy formulations.

In Figure 5, we present loss moduli of final materials. As expected, the peak temperature rises with increasing epoxy content, suggesting good compatibility between the polyacrylate and the copolyester networks. However, it is observed that with an epoxy content of 60 wt.% (E40DGMA60), a second relaxation peak at higher temperature is observed, suggesting phase separation. As this second peak converges to the peak of pure DGMA with increasing epoxy content, one can speculate that this phase is rich in epoxy–anhydride polymer, while the first one corresponds to a phase richer in the polyacrylate network, in parallel with previous reports [17].

**Figure 6:**
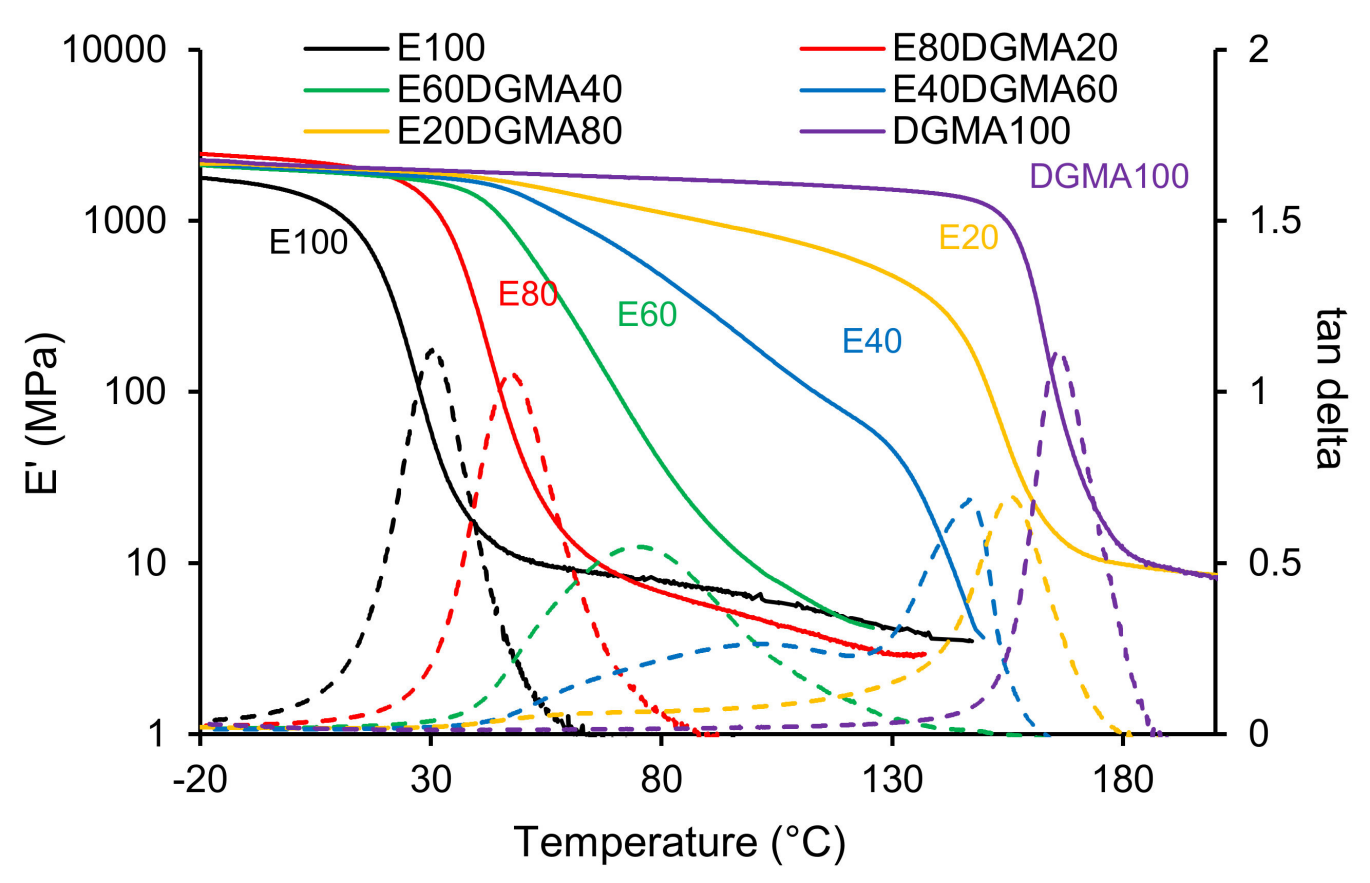
Storage modulus and tan delta (dashed) curves of all ExDGMAy final materials.

The existence of two network relaxations is more evident from the storage modulus and tan delta curves given in Figure 6. A similar analysis follows, with the relaxation temperatures converging to that of the pure DGMA, although not as much as the loss modulus peaks, as the dual materials’ DGMA content increases. The alpha relaxations also shift to higher temperatures, suggesting that polyacrylate and epoxy–anhydride networks are not completely immiscible. The increase in width, accompanied by the decrease in height of the primary relaxations, imply an increase in crosslinking density and a decrease in network homogeneity, as epoxy–anhydride content is increased.

As can be seen, depending on the formulation, the alpha relaxation temperatures range between 40 to 180 °C. The results suggest that with 40% addition of epoxy–anhydride mixture to the commercial acrylate resin, after complete cure, a glassy solid is obtained at ambient temperature (E60DGMA40). This is a stark contrast to the original unmodified acrylate resin (E100), which is an elastomer with poor mechanical properties. Any dual formulation presented here, when 3D printed and thermally treated, not only would have improved mechanical performance, but also reduced curing shrinkage due to reduced acrylate content [9].

#### 3.2.2. Network Topology and Phase Separation

**Figure 7:**
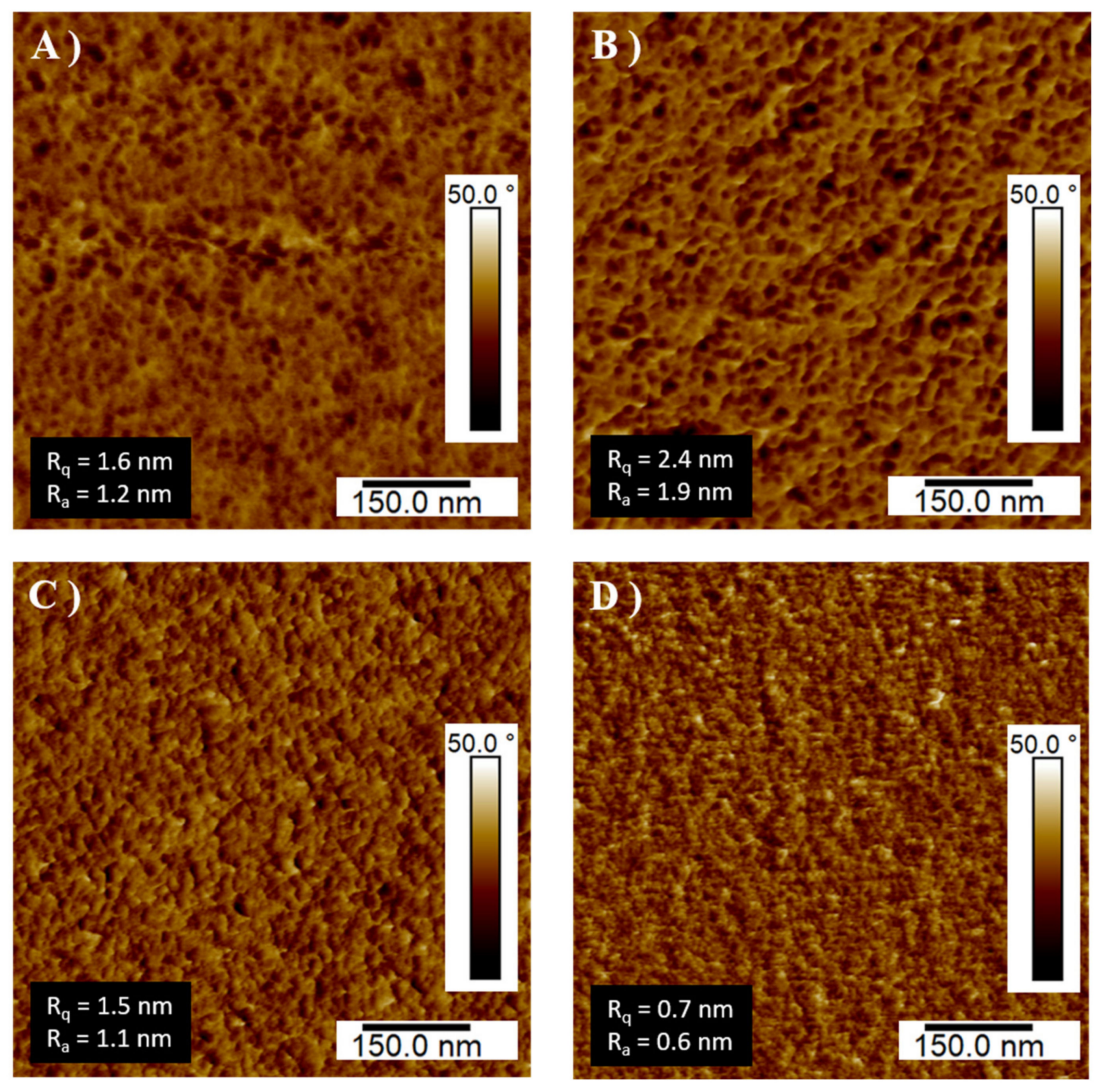
AFM phase images of E100 (**A**), E40DGMA60 (**B**), E32.5DGMA52.5DGCA15 (**C**), and DGMA100 (**D**). The segregated nanodomains appear as black patches.

Figure 7 shows the AFM phase images of the different materials. There appears to be an intrinsic heterogeneity in the acrylate resin E100, which is aggravated in the dual formulation E40DGMA60. As can be seen in Table 2, the size of the segregated nanodomains grow from 9 ± 2 nm in E100 to 17 ± 4 nm in E40DGMA60. In Figure 7, the phase image of the pure epoxy–anhydride DGMA100 is also shown. Its topology suggests this material is the most homogeneous among the set. The surface roughness, calculated as roughness average (R_a_) and root mean square roughness (R_q_) of each sample, is indicated at the bottom-left of each image. The calculation method is described in an earlier paper [33]. The values follow the same trend as nanodomain sizes, thereby supporting our claims about phase separation.

Following a similar procedure as in our previous work [17], using the data in Figure 4, we estimated the composition of the different phases in the materials E20DGMA80 and E40DGMA60. The results are summarized in Table 3.

The very low presence of a polyacrylate network in the high-*T_g_* (i.e., hard) phases (composed predominately of copolyester network) is not unexpected given that the polyacrylate network is already formed before the epoxy–anhydride copolymerization starts. As a means of comparison, we can consider E60DGMA40. Although the presence of a second phase is not evident in this formulation, had there been phase separation, the main (soft) phase would be 69.5% rich in the polyacrylate network, and it would constitute >80% by wt. of the material, which explains why the contribution of a second, epoxy-rich high-*T_g_* phase is not evident from the DMA data. Taking all this into consideration, the solubility threshold of the second phase appears to be between 20% and 40% by weight of the DGMA component.

**Figure 8:**
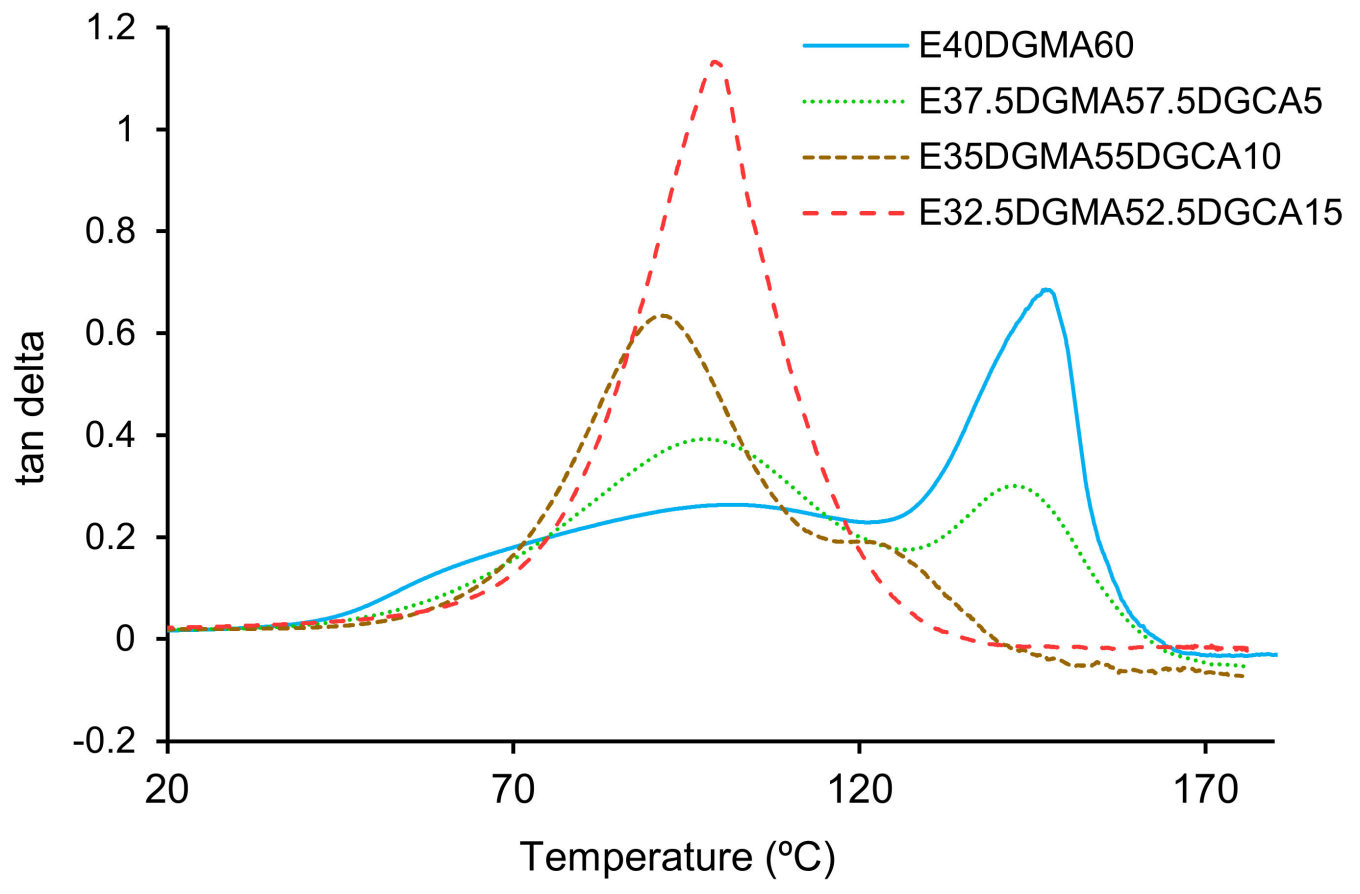
Tan delta curves of E40DGMA60 and its CA containing counterparts.

Even though phase separation occurs at the nano-scale, with no apparent turbidity in the final materials, it can complicate the precise definition of material properties and/or jeopardize material performance and therefore be undesirable in some applications. For interpenetrating networks exhibiting strong phase separation, if one phase is inherently brittle, the resulting material might suffer from low toughness. In those cases, covalently linking the two networks might facilitate effective dissipation of impacts (given a higher toughness of the other phase), thereby increasing material toughness, as was described by other researchers [34]. Moreover, one may desire a unimodal relaxation profile, so that the relaxation temperature range is able to shift upon changing the composition. Note that in phase separated samples with different compositions, the secondary relaxation temperature range seems confined between 130 and 180 °C (see Figure 6). The phase separation can be effectively mitigated by the help of a coupling agent (CA), as was already suggested before. The coupling agent used in this work is a carboxylic acid, namely 2-carboxyethyl acrylate, which bears an acrylate end group, and thus it can be incorporated into both networks, as previously described (see Scheme 2). When preparing formulations with CA, it was assumed that the number of epoxy groups would be equal to the sum of carboxylic acid groups and methacrylate groups. Thanks to its dual acrylate and acid functionality, it can be incorporated into both networks during their individual polymerizations. For these formulations, coded as ExDGMAyDGCAz, it can be observed from Figure 8 that as DGCA content increases, the secondary peaks decrease in intensity, until complete disappearance at a DGCA content of 15%. The compatibilizing effect of the coupling agent is also evident from the AFM phase images given in Figure 7. In comparison to the E40DGMA60, nanodomain size (see Table 2) and surface roughness are significantly lower in E32.5DGMA52.5DGCA15, suggesting increased homogeneity of this material.

In choosing the optimal formulation for 3D printing, we resort to Figure 4, as it summarizes the whole range of plausible *T_g_* in a reader-friendly way, and it can be used to determine adequate compositions for the intended application. For example, if one desired to obtain a rubbery material after the first curing stage that would later cure to a glassy solid with *T_g_* above 70, they would have to choose an acrylate component weight fraction lower than 0.4. In addition, the *T_g_* of the intermediate materials reported in Figure 4 and the inherent flexibility of neat E100 material suggest that these intermediate materials are ductile. Indeed, a few trials revealed that E40DGMA60 provided a good balance between shape consistency at the intermediate stage and high *T_g_* at the final stage. This allows one to impose complex shapes on the 3D printed, partially-cured, and ductile parts, and permanently fix them in the subsequent thermal cure step, as it is commonly done with other dual-curing systems [35]. In the context of 3D-printing, this could be exploited in order to save printing time as the geometry at intermediate stage can be chosen such that the number of printed layers is minimized. The printed, partially-cured and ductile (i.e., intermediate stage) material could be shaped as desired, and the shape can be consolidated after the completion of the second curing stage. An example will be provided in the following sections.

With regard to phase separation, even though for certain applications it would be undesirable, for certain others that require good performance at high temperatures, it might be beneficial. For example, E40DGMA60 at the fairly high temperature of 120 °C would still retain significant mechanical strength, since its secondary phase would still not have relaxed yet. Had there been no phase separation, the material would have relaxed completely, since its *T_g_* would be below 120 °C, as estimated by the Fox expression (blue line on Figure 4).

#### 3.2.3. Mechanical Properties

**Figure 9:**
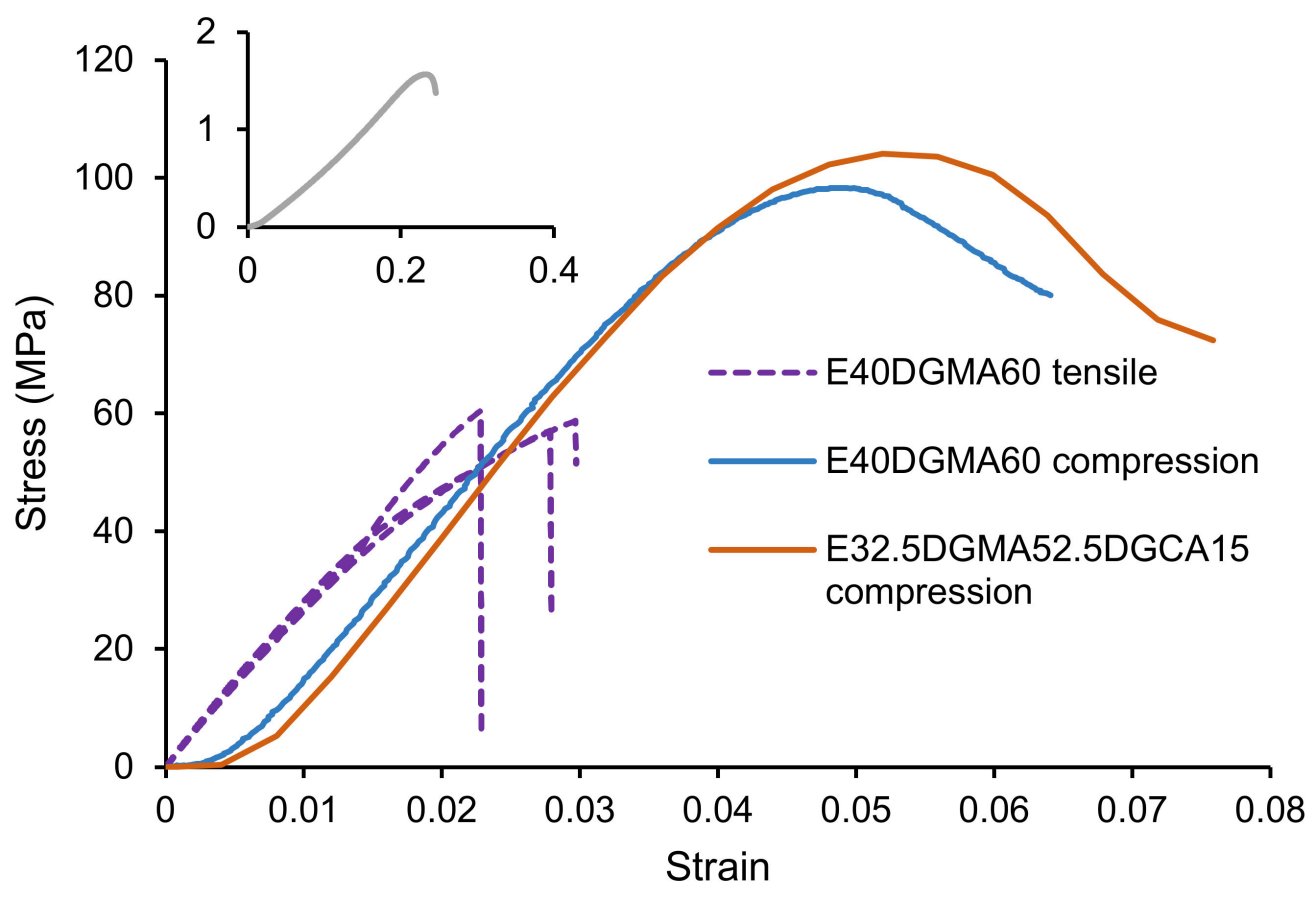
Compression stress–strain curves of E40DGMA60 and E32.5DGMA52.5DGCA15 contrasted to tensile stress–strain curves of E40DGMA60. Inset: Compression stress–strain curve of E100.

Compression and tensile tests performed on the dual formulation E40DGMA60 revealed significant improvement of mechanical performance over the neat acrylate, as expected. Results are summarized in Figure 9. Whereas the pure E100 material was essentially a soft rubber with a storage modulus slightly below 7 MPa in compression tests (see inset of Figure 9), E40DGMA60 was a rigid solid with an average storage modulus of 2.87 GPa and 2.98 GPa, calculated from compression test and tensile test results, respectively. We only used the initial region of elastic behavior, where the stress response of the sample was fairly linear, to calculate the moduli. In compression tests, we used the linear region between strain values of 0.01 and 0.02 (corresponding to tension values of 20 to 40 MPa, approximately) to calculate the moduli. Another result, although of secondary importance, is that the E40DGMA60 material seems to withstand compressive load better than tensile load. The formulation with CA (E32.5DGMA52.5DGCA15) was virtually undistinguishable from E40, suggesting that CA did not have any effect on compression behavior.

### 3.3. Prospective 3D Printing Applications

**Figure 10:**
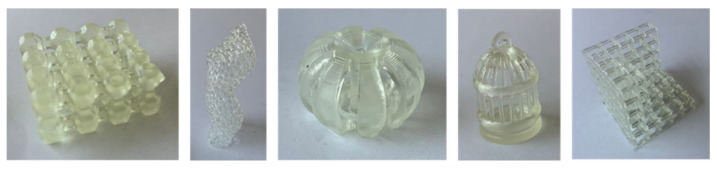
Complex-shaped objects printed from E40DGMA60. No thermal treatment is applied. From left to right: zeolite structure, fish-net woman, fountain light, bird cage, perforated cube (staggered).

As with any photocure-based additive manufacturing technique, complex shaped objects with intricate details can be easily and rapidly printed in our DLP printer using the E40DGMA60 formulation. Some examples are given in Figure 10. In the printing of all these objects, the layer thickness was 100 μm and each layer received an irradiation of 17 mJ·cm^−2^.

In addition to these decorative applications, the two-stage preparation procedure proves handy in certain other contexts, as will be detailed in what follows.

#### 3.3.1. Shape Memory Cube Printed at Reduced Cost

**Figure 11:**
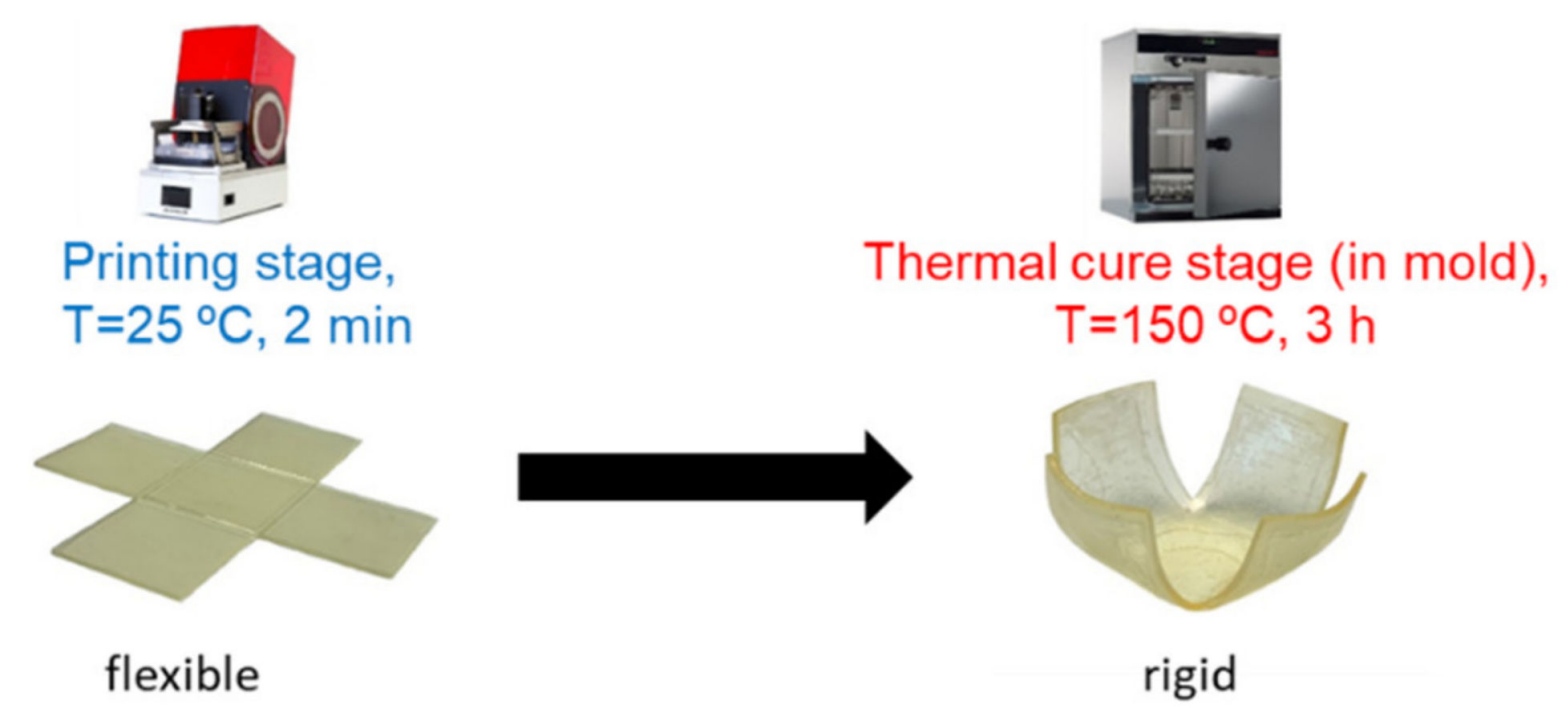
Cost-effective manufacture of the shape memory material.

Using the E40DGMA60 formulation, we first printed a cross shaped object with the DLP printer, which took less than 2 min. As the printed material was partially cured, it had low *T_g_* and was highly flexible. We placed this material into a custom-designed mold to bend its sides and thermally cure it at 150 °C for 3 h to obtain the final material with a “tennis ball holder” shape. The preparation scheme is depicted in Figure 11. This print geometry would significantly reduce manufacturing costs, since the number of printed layers is minimized. It would also eliminate the need for extraneous supporting structures during the printing process.

**Figure 12:**
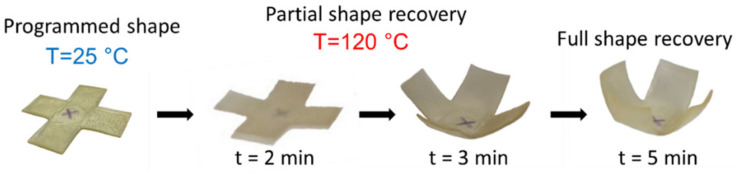
Shape programming and recovery of the E40DGMA60 “tennis ball holder”.

The glass transition profile of E40DGMA60 renders it suitable as a shape memory material. The property of shape memory is not intrinsic to any polymer. Through thermomechanical manipulations, the material is programmed into an unstable, temporary shape. This temporary shape is stabilized by strong interactions that impede mobility of the polymer chains, such as when the material is cooled down below its *T_g_* [36]. Once the material is heated above its *T_g_*, the mobility restrictions are lifted, and it returns to its stable state (original shape). The fully cured material was mostly relaxed at 150 °C, so it is possible to program a temporary, flat shape (same as the original cross shape), as shown in Figure 12. Once cooled down below its network relaxation range, the programmed shape can be fixed. When heated up to a moderate 120 °C, the recovery commences. As can be seen in Figure 12, full recovery was observed in 5 min. The shape fixity and shape recovery can be quantitatively characterized as future work. Nevertheless, we can safely conclude that the E40DGMA60 material can be programmed into any temporary shape, as required by transport or processing conditions, only to recover the original shape once heated up to a sufficiently high temperature to allow network relaxation.

#### 3.3.2. Production of Large Components by Parts

**Figure 13:**
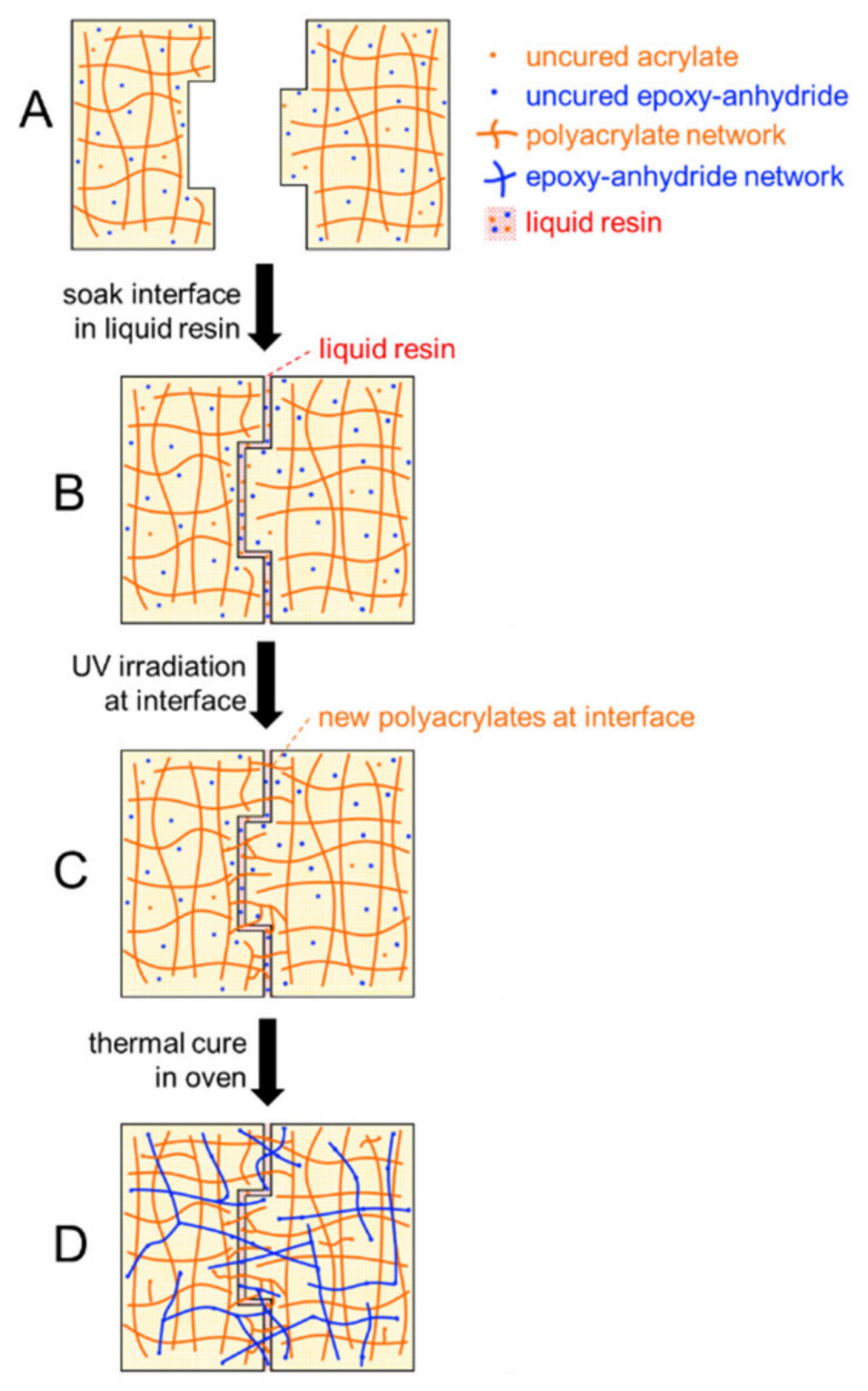
The union procedure for large part production. The printed halves (**A**) contain uncured acrylates and uncured epoxy–anhydride. By wetting the interface with the liquid resin, surface irregularities are filled, and good interfacial contact is ensured (**B**). Local UV irradiation at the interface promotes covalent bonding via acrylate photopolymerization (**C**), at which stage the adhesion is sufficiently strong to allow manipulation. The subsequent thermal cure contributes with more covalent bonds (via epoxy–anhydride copolymerization and residual acrylate polymerization) (**D**).

In dual-cure processing, 3D-printed parts can be bound covalently, hence permanently, by joining intermediate stage materials together and activating the second curing stage. This possibility was also outlined by other researchers using different dual-curing systems, albeit with the purpose of repairing damaged 3D-printed structures [24]. However, other applications of this adhesive behavior can be envisaged. When large parts (whose either dimension exceeds 10 cm) are considered, the small printing stages (or platforms) of home-size DLP printers present a limitation. One way to circumvent this is to adopt a modular approach, print the object in parts, and join them in a subsequent step. The procedure we adopted is schematized in Figure 13.

**Figure 14:**
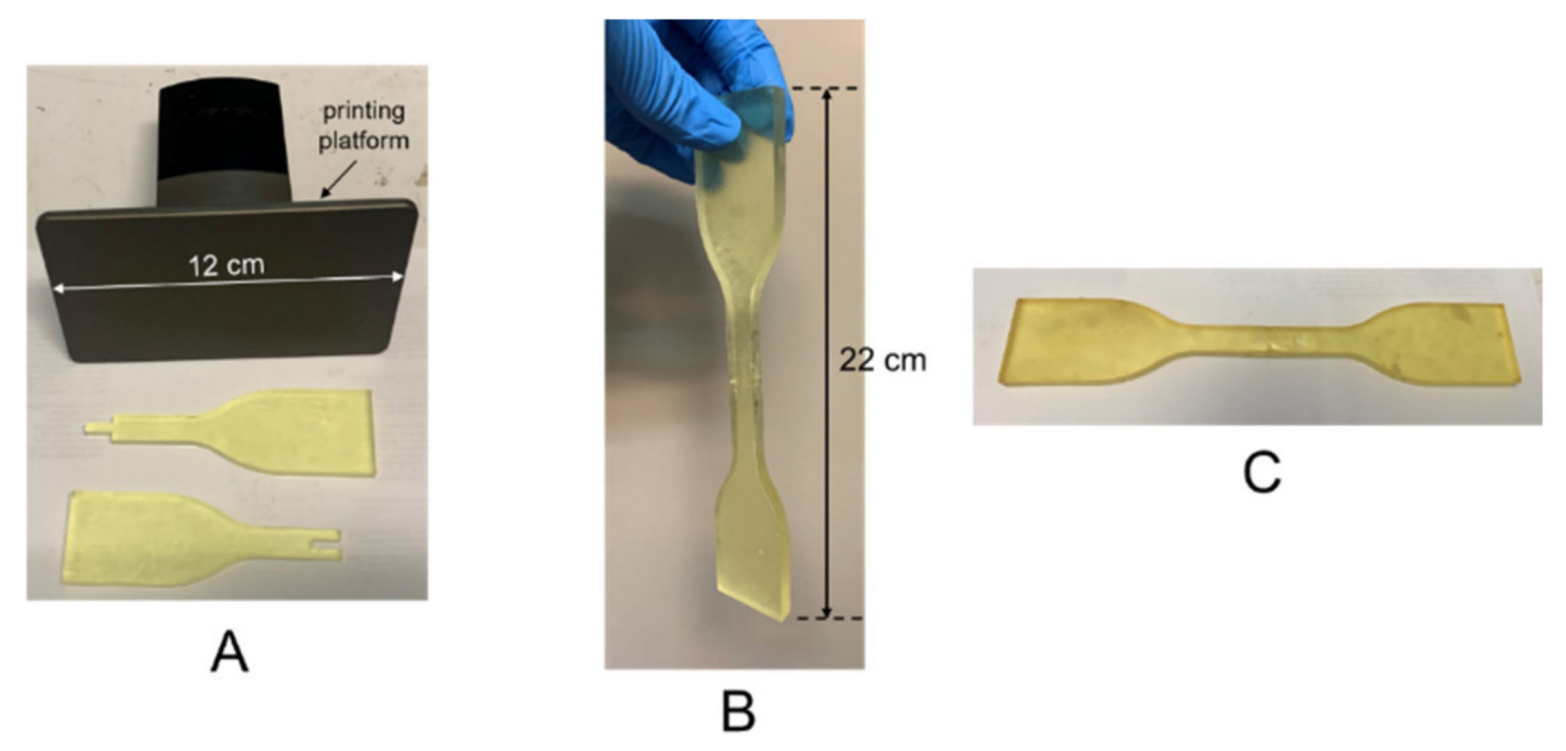
(**A**) The two halves of the dog-bone printed with maximum size allowable. (**B**) The soldered intermediate material withstands gravity. (**C**) The thermally treated, fully cured dog-bone with seamless union of its two halves.

**Figure 15:**
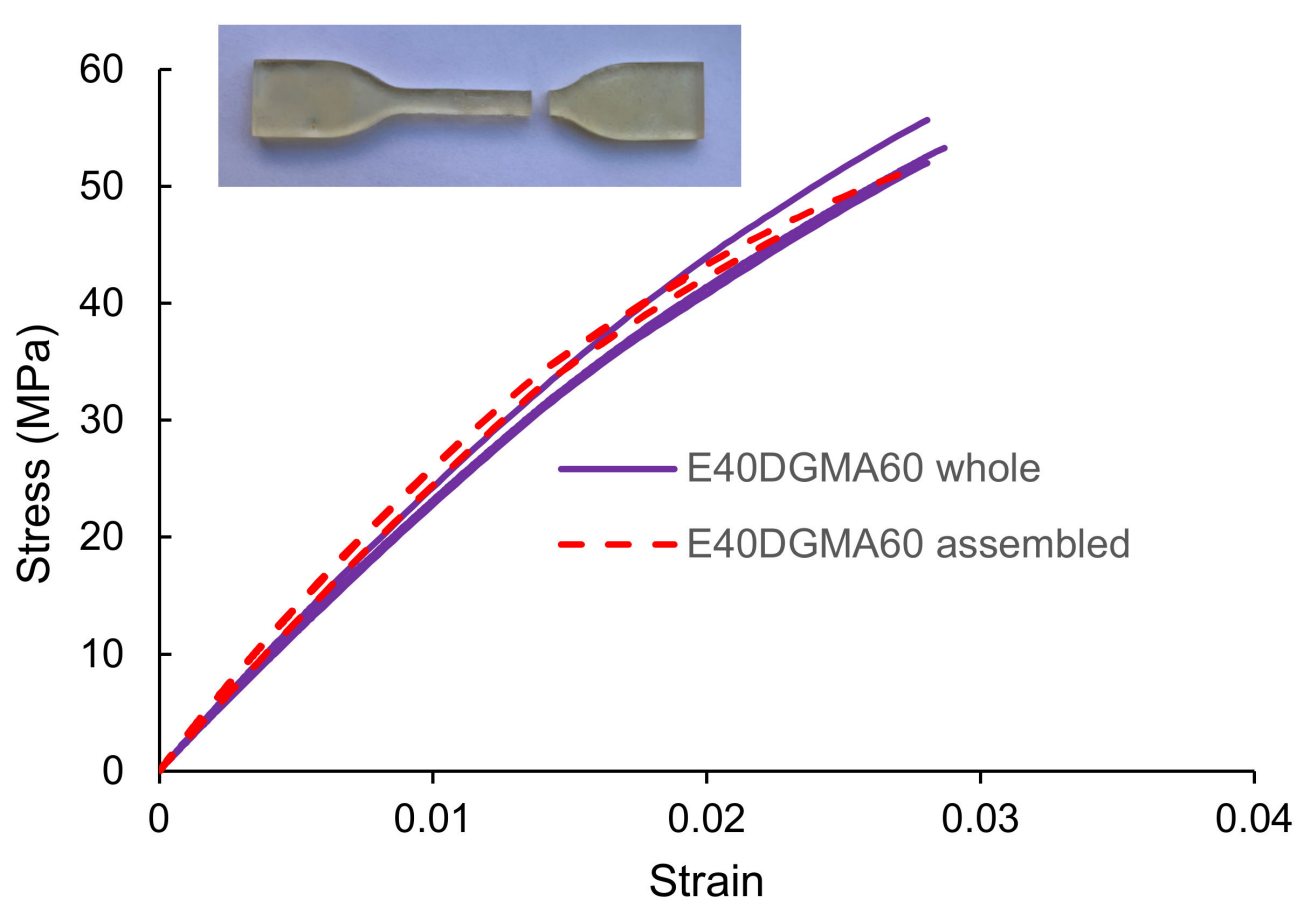
Tensile stress–strain curves of whole and assembled E40DGMA60 dog-bone specimens. Three whole and three assembled specimens were tested. Curves are truncated at fracture. Inset: Off-center fracture of an assembled specimen.

To demonstrate this procedure, we first printed the two halves of a larger dog-bone specimen, as seen in Figure 14A. The choice of the dog-bone geometry is arbitrary. We joined the two halves by the particular soldering method depicted in Figure 13: We soaked the union with the uncured, liquid formulation, wiped off the excess, joined the halves and irradiated the union locally using a UV lamp (Hamamatsu Lightningcure LC5 with a mid-pressure Hg–Xe UV lamp effective irradiation wavelength 360 nm, light intensity 23 mW/cm^2^). As can be seen in Figure 14B, after this soldering, adhesion was already sufficient, but bond strength would only be limited, because this treatment only initiates the polymerization of uncured acrylates, either remaining from the 3D-printing step or contained in the uncured resin used to wet the union. After a thermal cure at 150 °C for 3 h, reaction of the epoxy fraction led to a much stronger bonding, producing a final material, with virtually flawless union of its two halves (Figure 14C). The Young’s moduli of standard sized dog-bone specimens assembled in the same fashion showed no practical difference compared to whole dog-bone specimens, as can be seen in Figure 15. The ultimate strengths and failure modes of both assembled and whole specimens were similar and highly repeatable, with one assembled specimen exhibiting slightly lower ultimate strength. The specimens failed by fracture at off-center points, as shown in the inset of Figure 15. All in all, the results confirm the success of the union procedure.

## 4. Conclusions

We presented a versatile method to obtain DLP-3D printed parts with good mechanical performance and shape memory capability. The use of a hybrid acrylate–epoxy formulation yielded flexible and ductile materials after printing, which were easily manipulated into complex shapes that could be fixed in a subsequent thermal curing stage.

The phase separation that resulted could be controlled by the help of a coupling agent. Whereas a phase separated material might perform well at high temperatures without compromising its rigidity, a more homogeneous network structure might be crucial for other applications requiring narrow glass transition ranges, such as in stimuli responsive materials. Despite the nanoscale phase separation that bestowed the dual materials with a complex network relaxation profile, temporary shapes could be programmed with fairly fast recovery to the original shape upon heating mildly above their primary relaxation temperature (i.e., the relaxation temperature of the acrylate-rich phase).

As was expected, the epoxy–anhydride component contributed greatly to mechanical performance, manifested as Young’s modulus increases of several orders of magnitude over the neat acrylate resin.

The partially-cured intermediate materials could be assembled into larger objects by employing a simple procedure that ensures covalent linking and apparently flawless interfaces after the second curing stage. Multi-material designs that mimic biological functions can draw inspiration from this latter approach.

## Figures and Tables

**Table 1:** Composition of the different formulations prepared in this work. In all cases, 0.25 phr of LUP with respect to the Spot-E content and 0.025 phr of DMAP with respect of the DG-MA content were used.

Formulation	E (wt.%)	CA (wt.%)	DG (wt.%)	MA (wt.%)
E100	100	0	0	0
E80DGMA20	80	0	10.48	9.52
E60DGMA40	60	0	20.96	19.04
E40DGMA60	40	0	31.44	28.56
E20DGMA80	20	0	41.93	38.07
DGMA100	0	0	52.38	47.56
E37.5DGMA57.5DGCA5	37.5	2.39	32.73	27.37
E35DGMA55DGCA10	35	4.79	34.02	26.18
E32.5DGMA52.5DGCA15	32.5	7.18	35.32	24.99

**Table 2:** Sizes of nanodomains seen in AFM phase images of Figure 7.

Formulation	Nanodomain Size (nm)
E100	9 ± 2
E40DGMA60	17 ± 4
E32.5DGMA52.5DGCA15	10 ± 2
DGMA100	7 ± 2

**Table 3:** Phase composition and content of dual formulations given as weight fractions.

Formulation	w_soft_	w_hard_	w_acrylate,soft_	w_acrylate,hard_
E20DGMA80	0.336	0.664	0.467	0.065
E40DGMA60	0.566	0.434	0.604	0.134

## Data Availability

The data are available from the authors upon request.
